# Security Framework for Network-Based Manufacturing Systems with Personalized Customization: An Industry 4.0 Approach

**DOI:** 10.3390/s23177555

**Published:** 2023-08-31

**Authors:** Muhammad Hammad, Rashad Maqbool Jillani, Sami Ullah, Abdallah Namoun, Ali Tufail, Ki-Hyung Kim, Habib Shah

**Affiliations:** 1 Faculty of Mechanical Engineering, GIK Institute of Engineering Sciences and Technology, Topi 23640, Pakistan; 2 Faculty of Computer Science and Engineering, GIK Institute of Engineering Sciences and Technology, Topi 23640, Pakistan; rjillani@giki.edu.pk; 3 Department of Computer Science, Shaheed Benazir Bhutto University, Sheringal 18050, Pakistan; sami@sbbu.edu.pk; 4 Faculty of Computer and Information Systems, Islamic University of Madinah, Madinah 42351, Saudi Arabia; a.namoun@iu.edu.sa; 5 School of Digital Science, Universiti Brunei Darussalam, Tungku Link, Gadong BE1410, Brunei; ali.tufail@ubd.edu.bn; 6 Department of Cyber Security, Ajou University, Suwon 16499, Republic of Korea; kkim86@ajou.ac.kr; 7 Department and College of Computer Science, King Khalid University, Abha 62529, Saudi Arabia; hurrahman@kku.edu.sa

**Keywords:** Industry 4.0, smart manufacturing, newly personalized customization factory, network-based manufacturing system, mobile industrial robots, security, NTRUEncrypt cryptography

## Abstract

Smart manufacturing is pivotal in the context of Industry 4.0, as it integrates advanced technologies like the Internet of Things (IoT) and automation to streamline production processes and improve product quality, paving the way for a competitive industrial landscape. Machines have become network-based through the IoT, where integrated and collaborated manufacturing system responds in real time to meet demand fluctuations for personalized customization. Within the network-based manufacturing system (NBMS), mobile industrial robots (MiRs) are vital in increasing operational efficiency, adaptability, and productivity. However, with the advent of IoT-enabled manufacturing systems, security has become a serious challenge because of the communication of various devices acting as mobile nodes. This paper proposes the framework for a newly personalized customization factory, considering all the advanced technologies and tools used throughout the production process. To encounter the security concern, an IoT-enabled NBMS is selected as the system model to tackle a black hole attack (BHA) using the NTRUEncrypt cryptography and the ad hoc on-demand distance-vector (AODV) routing protocol. NTRUEncrypt performs encryption and decryption while sending and receiving messages. The proposed technique is simulated by network simulator NS-2.35, and its performance is evaluated for different network environments, such as a healthy network, a malicious network, and an NTRUEncrypt-secured network based on different evaluation metrics, including throughput, goodput, end-to-end delay, and packet delivery ratio. The results show that the proposed scheme performs safely in the presence of a malicious node. The implications of this study are beneficial for manufacturing industries looking to embrace IoT-enabled subtractive and additive manufacturing facilitated by mobile industrial robots. Implementation of the proposed scheme ensures operational efficiency, enables personalized customization, and protects confidential data and communication in the manufacturing ecosystem.

## 1. Introduction

**Figure 1:**
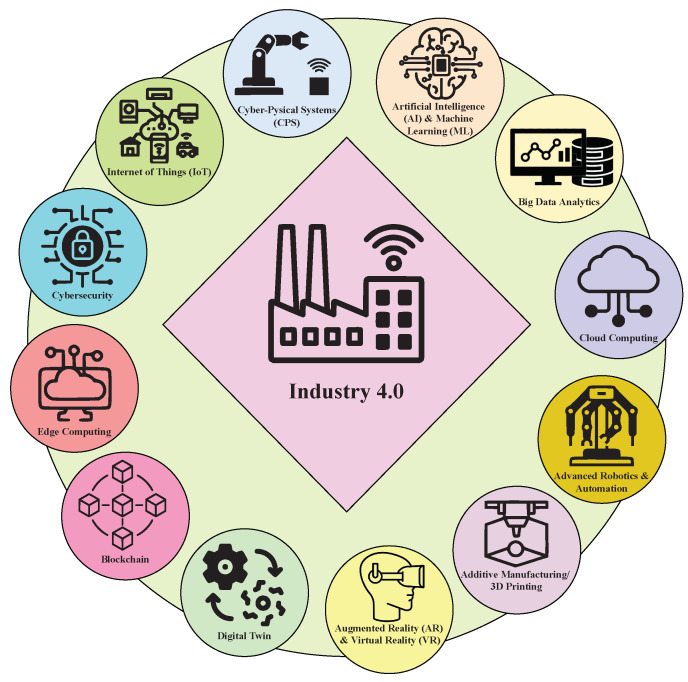
Components of Industry 4.0.

The Fourth Industrial Revolution (Industry 4.0) pertains to contemporary trends in automation and information exchange within manufacturing technologies. It encompasses various innovative technologies, including the Internet of Things (IoT), artificial intelligence, cyber-physical systems (CPSs), big data analytics, digital twin, cloud computing, additive manufacturing, etc., as shown in Figure 1. It leverages these technologies to transform traditional manufacturing into highly interconnected, smart manufacturing systems [1,2]. The primary aim of Industry 4.0 is to establish smart factories in which CPSs oversee physical operations, generate digital replicas of the tangible environment, and take actions rooted in real-time information. The significance of Industry 4.0 lies in the interconnected systems, increased efficiency, personalized customization, resource efficiency, and safety and security [3,4]. As IoT technology becomes a prominent field of communication, the scope of its applications is expanding into the industrial sector. Hence, the novel approach of connecting industrial equipment to the internet has led to an extension of the IoT called Industrial IoT (IIoT). Thus, IoT and IIoT are integral and pivotal components of the Industry 4.0 paradigm, offering less expensive, robust, and practical solutions to manufacturing paradigms. IoT comprises interconnected computing devices, such as electronic systems, objects, and living organisms that are capable of communicating data across a network without requiring human or human–machine interaction [5]. Similarly, IIoT is a subset of the broader concept of the IoT but tailored specifically for industrial environments. It refers to the interconnection of industrial equipment, devices, and systems to collect, exchange, and analyze data to optimize industrial and manufacturing processes [6]. Linking diverse technological platforms offers the capability to interact with remotely situated manufacturing machinery, regardless of the user’s geographical location. Consequently, product designers can transmit their product designs for manufacturing to a broader range of potential production facilities. This evolution within the manufacturing sector results in a more adaptable yet finely tuned production setting, presenting a flexible and optimized manufacturing environment.

IoT is changing the lives of humans and making them more comfortable [7,8,9]. IoT communications take place with a set of predefined models and protocols, such as ad hoc on-demand distance vector (AODV) routing, destination-sequenced distance vector vector (DSDV) routing, optimized link state routing (OLSR), dynamic source routing (DSR), routing protocol for low power and lossy networks (RPL), constrained application protocol (CoAP), and 6LoWPAN (Ipv6 over low-power wireless personal area networks). IoT communications occur in four layers, i.e., the perceptual layer, support layer, application layer, and network layer. The IoT nodes can connect with their neighbors and proceed with data among other nodes [10,11,12,13]. Thus, the IoT plays a tremendous role in the manufacturing sector, in regard to enabling smart manufacturing. In addition, IoT-enabled mobile industrial robots (MiRs) are significantly contributing to smart manufacturing by leveraging IoT to enhance their capabilities. By collecting real-time data, performing predictive maintenance, navigating autonomously, collaborating with human operators, and enabling remote monitoring and control, these robots are helping manufacturers improve efficiency, reduce costs, and enhance product quality. MiRs are designed to be flexible and easily programmable, so they can be adapted to different tasks and workflows. They can be programmed to enable smart manufacturing, e.g., by transporting materials from one location to another, picking up and delivering materials to specific workstations, or assisting with inventory management tasks [14,15]. Having established the profound role of IoT and its associated communication protocols, particularly in harnessing the potential of MiRs for a truly manufacturing ecosystem, it is imperative to delve deeper into the overarching structures that guide and govern this digital integration. This leads to the concept of Industry 4.0 framework models. With the rapid advances in technology, new concepts such as Reference Architecture Module Industry 4.0 (RAMI 4.0) and Asset Administration Shell (AAS) have emerged in the Industry 4.0 framework.

RAMI 4.0 is a comprehensive, three-dimensional conceptual framework that provides a structured approach to understanding and implementing Industry 4.0 concepts. It acts as a reference for companies to digitize their processes in line with Industry 4.0 principles. It ensures that all aspects of the industry, from business processes to technical infrastructure, are addressed. Its model includes hierarchical levels, a life cycle combined with a value stream, and various layers. Hierarchical levels entail product and field devices, control devices, stations, work centers, enterprises, and even connections between enterprises. The life cycle and value stream map the product’s idea from its inception to its end; it is aligned with IEC 62890. Layers break down the industrial system into various layers, such as the asset, integration, communication, information, functional, and business layers [16,17]. As Industry 4.0 connects a vast array of devices and systems, ensuring secure communications and operations is paramount. Security in RAMI 4.0 is addressed in the “information layer”. This layer ensures data integrity, confidentiality, and availability. It also emphasizes the importance of protecting data from unauthorized access, tampering, and breaches. Security considerations are embedded in each layer, from the field to the business level. A safety and security model based on RAMI 4.0 has been proposed to address the security challenges of Industry 4.0 [18,19]. AAS is a central idea in Industry 4.0, acting as a digital counterpart or a virtual depiction of a tangible asset. This concept functions as a uniform interface, guaranteeing that an asset’s data and capabilities are accessible across various platforms [20]. The AAS is crafted to function independently of specific platforms and manufacturers, necessitating its secure operation across diverse settings. Its security measures include authentication, ensuring data integrity, maintaining confidentiality, and regulating access control. Within Industry 4.0, two pivotal security approaches have been proposed: one that integrates both the safety and security reference architecture and another that emphasizes secure provisioning. These approaches form the cornerstone for developing secure and safeguarded applications rooted in AAS [21,22]. RAMI 4.0 and AAS are instrumental concepts driving the paradigm shift in smart manufacturing.

Smart manufacturing is a fully integrated and automated system used to meet the requirements of the smart industry [23]. However, further research is needed from the implementation perspective because only a few studies focus on network-based interconnection factories for the smart manufacturing implementation system (SMIS). The network-based interconnection factory is characterized by the interconnection and interoperability layer of the SMS [24]. A network-based manufacturing system consists of distributed manufacturing scenarios that cope with customized product needs. It also integrates the firm’s information to provide the required services with the help of which interoperability among industries can be acquired. In NBMS, the production mode shifts to make-to-order from make-to-stock, and job requests from customers are fully customized [25]. IoT-enabled manufacturing systems pose different security challenges, including malware and security, data privacy, and insider threats. Smart manufacturing systems are always vulnerable to various types of network layer attacks. Denial of service (DoS) attacks are common in network-based collaborative industries. In a DoS attack, the attacker makes the machine or network unavailable to the authentic user by intruding into the network. DoS attacks are basically characterized by flooding the intended IoT network device with a bundle of requests. These requests encumber the resources and result in the unavailability of authorized users. The black hole attack is a common type of DoS attack where an attacker uses a compromised node in a network to attract and drop all packets destined for a specific destination or range of destinations, effectively creating a “black hole” in the network where data go missing or are never delivered to the destination. This type of attack can disrupt communications and compromise the integrity and availability of the network. A DoS attack is converted to a distributed denial of service (DDoS) attack if the flooding of request messages comes from multiple sources [26,27,28].

### 1.1. Research Gap

As manufacturing industries move toward the IoT, prioritizing operational security is necessary. Researchers must develop the framework for the implementation of security in smart manufacturing systems. Zhang et al. [29,30] developed a three-dimensional framework for SMS. They extended each layer of the SMS and SMIS. They also proposed a smart service system framework to enable personalized customization. Moreover, the authors of [30] significantly enhanced the product–customer interaction by providing an optimized filtering algorithm. However, these studies still require a security dimension, regarding the implementation of smart manufacturing.

### 1.2. Novelty and Contributions

The major contributions of this work are as follows:

- We propose a framework for SMIS that incorporates a newly personalized customization factory.
- We propose a secure IoT-enabled NBMS against the black hole attack, using NTRUEncrypt cryptography and the AODV routing protocol.
- We enable manufacturing firms to implement secure IoT-enabled subtractive and additive manufacturing via MiRs.
- We simulate the network using NS2 to evaluate three different networks: the healthy network, malicious network, and an NTRUEncrypt-secured network.

### 1.3. Paper Organization

The rest of this article is organized as follows: Section 2 presents related work, Section 3 presents a framework for SMIS and the simulation networks of NBMS. Section 4 presents evaluation metrics to evaluate three different networks, including a healthy network, a malicious network, and an NTRUEncrypt-secured network. Section 5 presents the results and discussion. Lastly, Section 6 summarizes the findings.

## 2. Related Work

Various techniques have been described in the literature to enhance the efficiency and security of IoT-enabled systems by utilizing appropriate protocols and algorithms. Jinhui et al. [31] introduced an intrusion detection technique with an appropriate algorithm to detect the hybrid DoS attack in a wireless network. Experiment results showed that the proposed algorithm could enhance the detection rate of attacking nodes, increase the network’s lifetime, and reduce the attack’s impacts on network traffic. However, this method is based on the very high precision of nodes for controlling their energy consumption. If the attacker controls the node fully and copes with the energy information, the malicious node escapes from protection. Kalkha et al. [32] developed an algorithm based on the hidden Markov model (HMM) to tackle the black hole attack in a wireless network by providing the optimal path. The NS2 simulator is used under the AODV protocol to evaluate metrics such as the packet delivery ratio (PDR), end-to-end (E2E) delay, and packet drop ratio. The simulation has shown efficient results for a secure network. Babaeer et al. [33] introduced a lightweight and secure technique to tackle the sinkhole attack in the wireless network. A sinkhole attack is a network layer attack where an attacking node attracts other nodes and deceives them by showing itself as the optimal path to the corresponding station. The proposed technique shows better results while considering the performances of the following: the packet delivery ratio, delay, energy consumption, and throughput. Bhosale et al. [34] proposed the intrusion detection system (IDS) to detect the network layer attack called the wormhole attack in IoT networks using the RPL protocol. The scheme was implemented in Cooja and Contiki simulators. The results identified the attacker node and attack in the network. The limitation of this work was the limited node numbers, i.e., 24 nodes. Ding et al. [35] developed an algorithm to inspect selective forwarding attacks in the wireless network. This attack dropped many packets when the data were received at the destination. The algorithm also reduced useless steps in density peak clustering to enable better detection accuracy. The simulation showed that the false detection rate lies below 1%, which is tremendous for the algorithm’s accuracy. Hashemi et al. [36] proposed the backdoor technique to enable penetration testing. Penetration testing is beneficial for locating IoT backdoors, detecting hacker action, preventing hackers from gaining permanent, concealed, and unauthorized access, penetrating testing aids in the detection of IoT backdoors, detecting attacker activity, and preventing them from gaining unauthorized access. Ezhilarasi et al. [37] secured the IoT networks from multiple network layer attacks using fuzzy- and feed-forward neural networks. Experimental results showed that the scheme has an average detection rate of 97.8%. The performance of this scheme is better than others but computation times are high compared to machine learning techniques. Li et al. [38] researched and analyzed the privacy difficulties involving data owners, untrustworthy third-party cloud servers, and data consumers under the IoT, and suggested a lightweight privacy-preserving technique based on homomorphic encryption. Meanwhile, computationally efficient homomorphic methods protect data consumer privacy. According to experimental results, the suggested system successfully prevents privacy intrusions in IoT. Mohsin et al. [39] developed a data-driven framework to present a clear demarcation of IoT configurations and assess different IoT threats. It has much importance regarding security concerns and is collectively called the IoT checker. Hussein et al. [40] proposed a secure technique to manage and distribute the keys for IoT-based wireless sensor networks. They used the elliptic curve cryptography technique and the enhanced LEACH protocol under the parameters of dead nodes, network lifespan, and energy consumption. Goel et al. [41] proposed the lightweight encryption and OBT-based authentication technique (LEOBAT) based on lightweight cryptography to authenticate the required device in the IoT network using a unique ID. By doing so, the IoT network can be secured from various attacks. This technique is also compared with existing techniques, such as DES and blowfish, to claim efficient and fast authentication. Bilal et al. [42] introduced the intrusion detection model to secure the communication of IoT networks against different sinkhole attacks. The proposed model provided exceptional results, with a detection rate of 95%. Li et al. [43], considering the end-to-end route, developed a cluster-based algorithm for wireless mesh networks connected with the IoT to reduce the end-to-end delay and enhance the quality of the internet. Hammad et al. [44] proposed a mutual authentication and key agreement scheme to increase the security of the smart manufacturing industry against different attacks. The proposed scheme uses bitwise XOR operations with physical unclonable function (PUF), along with the elliptic curve cryptography (ECC) and cryptographic hash functions. Their work showed better results compared to the state-of-the-art schemes. An et al. [45] proposed a scheme that addresses the opacity problem of distributed estimation. The scheme employs the final-state opaque (FSO) technique to find opacity-enhancing distributed estimation while maintaining high accuracy. Moreover, it proposed consensus-based distributed estimation algorithms based on the bias-adding and state decomposition methods to provide the FSO in the presence of crafty intruders and malicious nodes, respectively. Lu et al. [46] devised a distributed optimization algorithm using homomorphic encryption. In this algorithm, two parties compute the objective value without showing their respective data. The algorithm also highlights the problem of input and output inference for a class of quadratic joint functions. So far, different studies have been performed to enhance the efficiency and security of IoT-enabled systems by utilizing appropriate techniques to tackle different network layer attacks. Table 1 presents a comparison of different eminent–existing related works.

## 3. Methodology

This section describes the proposed framework for a newly personalized customization factory and NBMS. The section also consists of the system model, network model, the effect of black hole attack in the IoT-enabled network, NTRUEncrypt-secured IoT-enabled NBMS, simulation setup, and experimental model.

### 3.1. Framework for SMS and SMIS

**Figure 2:**
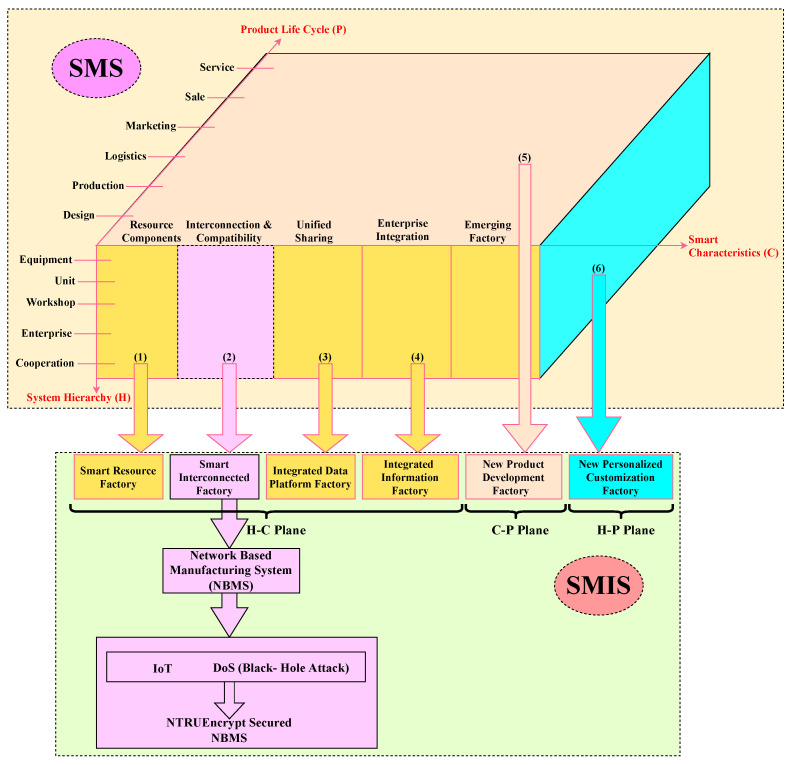
Framework for the SMS and SMIS.

The framework of the SMS consists of three chains, i.e., smart characteristics (C), system hierarchy (H), and product life cycle (P), as illustrated in the top section of Figure 2. The “Product Life Cycle” chain encompasses all activities involved in the product’s journey, from its initial design to its eventual disposal. The “System Hierarchy” chain pertains to the categorization of production resources and activities, ranging from individual equipment to collaborative operations. The “Smart Characteristics” chain consists of resource components, interconnection and compatibility, unified sharing, enterprise integration, and the emerging factory, reflecting the smart function. The integration of any two chains of the SMS generates the corresponding SMIS plane: the H-C plane is generated by the combination of “H” and “C” chains, similarly the C-P plane is formed by the integration of “C” and “P” chains, and the H-P plane is generated by the addition of “H” and “P” chains. Every plane has its layers. The H-C plane contains the smart resource factory, smart-interconnected factory, integrated data-platform factory, and integrated information factory. The C-P plane consists of the new product development factory and the H-P plane entails the newly personalized customization factory, as shown in the bottom section of Figure 2. The description of each terminology in the SMIS is as follows:

The smart resource factory utilizes advanced automation technologies to maximize resource efficiency and refine production processes. By automating multiple aspects of manufacturing, the factory can substantially improve effectiveness, diminish human mistakes, and reduce waste. In essence, a smart resource factory fosters heightened productivity, a competitive edge, and eco-friendliness in the manufacturing sector by optimizing resource allocation and capitalizing on the advantages of automation. The smart-interconnected factory leverages advanced networking technologies to create seamless communication and data sharing among machines, systems, and stakeholders throughout the production process. This interconnected network fosters real-time monitoring and control, enabling optimized manufacturing processes, improved decision-making, and increased efficiency. In essence, the smart-interconnected factory enhances productivity, flexibility, and competitiveness in the manufacturing industry by creating a highly connected and data-driven environment. The utilization of an integrated data-platform factory facilitates the acquisition of real-time insights, thereby enhancing the quality of decision-making, optimizing production processes, and augmenting overall efficiency throughout the factory. The platform-based data-sharing factory facilitates collaboration and transparency among stakeholders, enabling them to conveniently access, analyze, and exploit data, thereby promoting ongoing enhancement and novelty. The integrated information factory is a system that aims to establish a seamless connection between diverse information systems utilized in the production process, resulting in a unified and coherent data landscape. The implementation of an integrated information factory fosters collaboration and transparency in processes, resulting in ongoing enhancements, heightened flexibility, and improved resource alignment. A state-of-the-art manufacturing facility that employs advanced technologies and data-driven processes is commonly known as a new product development factory. This particular facility endeavors to optimize the potential of a given product throughout its complete life span. The implementation of advanced technologies in this factory, such as IoT, artificial intelligence (AI), and automation, facilitates efficient resource management, optimized production procedures, and enhanced decision-making capabilities throughout the entire product lifecycle, from design to disposal. The newly personalized customization factory employs modern techniques and tools to meet customer demands. It can manufacture the goods while considering the manufacturing environment’s flexibility and adaptability to meet the customer’s needs. It leads to maintaining a high level of efficiency and cost-effectiveness. By utilizing data analytics and instant feedback, the manufacturing facility can continuously alter and improve its production processes, producing goods that satisfy the customers’ needs.

#### 3.1.1. Framework for a Newly Personalized Customization Factory

**Figure 3:**
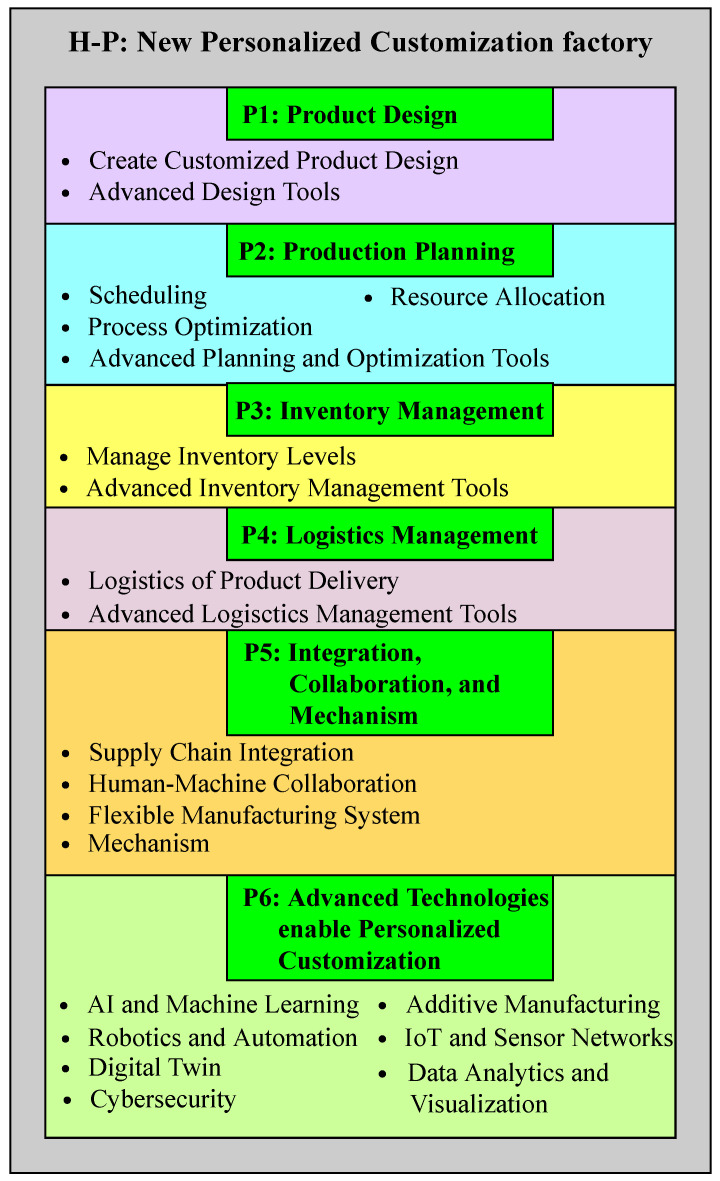
Framework for a newly personalized customization factory.

The system comprises six distinct layers, namely H-P1, H-P2, H-P3, H-P4, H-P5, and H-P6. H-P1 describes the product design, H-P2 pertains to production planning, H-P3 explains inventory management, H-P4 covers logistics management, H-P5 is related to integration and collaboration, and H-P6 comprises advanced technologies that facilitate personalized customization, as shown in Figure 3. In the product design layer, customized products are designed that meet customer requirements. It integrates advanced design and simulation tools that enable designers to create and test different product designs in a virtual environment. Production planning involves planning the production process, including scheduling, resource allocation, and process optimization. It leverages advanced planning and optimization tools that enable planners to create optimized production plans that balance customer demand, production capacity, and resource availability. Inventory management aids in managing inventory levels and ensuring that the right materials and components are available when needed. It leverages advanced inventory management tools that enable the real-time tracking of inventory levels and automated replenishment of materials and components. Logistics management is responsible for managing the logistics of the product delivery, including transportation and distribution. It leverages advanced logistics management and route optimization tools that enable the efficient and cost-effective delivery of products. The integration and collaboration layer consists of supply chain integration, human–machine collaboration, and a flexible manufacturing system; they collectively contribute to personalized customization. Advanced technologies that incorporate personalized customization include AI and machine learning, additive manufacturing, robotics and automation, IoT and sensor networks, a digital twin, data analytics, visualization, and cybersecurity.

**Figure 4:**
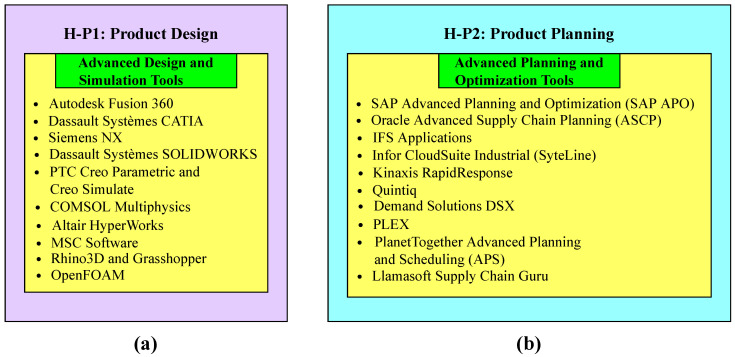
Extension of (**a**) H-P1 and (**b**) H-P2 layers.

**Figure 5:**
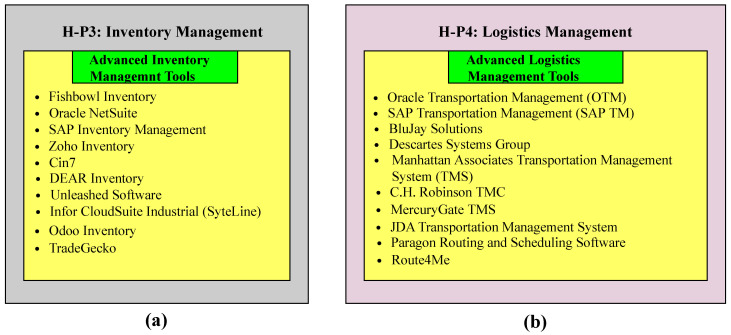
Extension of (**a**) H-P3 and (**b**) H-P4 layers.

**Figure 6:**
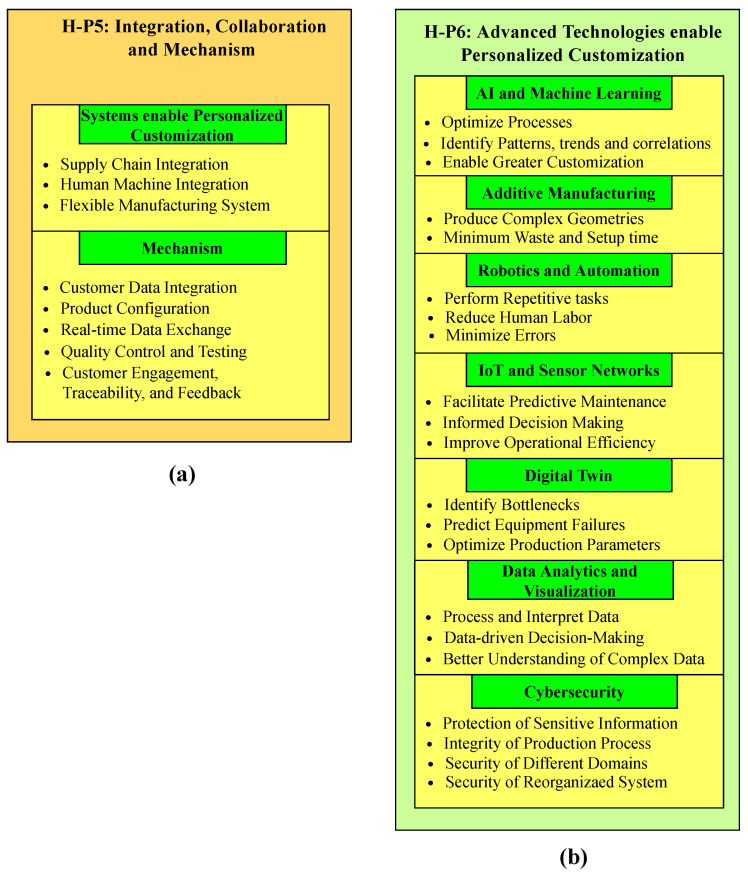
Extension of (**a**) H-P5 and (**b**) H-P6 layers.

1. Extension of the H-P1 layer.
  Advanced design and simulation tools play a prominent role in the product design process for a newly personalized customization process. The tools participating in this context are shown in Figure 4a. Autodesk Fusion 360 is a cloud-based CAD, CAM, and CAE tool that is used for the 3D modeling and manufacturing of products. It enables designers to test different design iterations, such as stress analysis, motion study, and thermal analysis. Dassault Systèmes CATIA is a leading CAD/CAM/CAE software that offers a comprehensive suite of design and simulation tools, enabling designers to create and test complex, customizable products for personalized manufacturing. Siemens NX is a computer-aided design (CAD)/computer-aided manufacturing (CAM)/computer-aided engineering (CAE) tool that helps designers to test and optimize the design for different parameters, such as strength, durability, and thermal performance. It accomplishes the required task by using advanced simulation functionalities. Dassault Systèmes SOLIDWORKS is a CAD package that assembles simulation capabilities to conduct various analyses under necessary factors. Finite element analysis (FEA), computational fluid dynamics (CFD), and fatigue studies are examples of these analyses. As a result, it is regarded as a reliable software option for product design. PTC Creo Parametric and Creo Simulate are CAD software programs that help designers create and modify intricate product designs by providing a wide range of simulation tools. Moreover, designers can conduct various assessments of products, such as structural, thermal, and vibrational properties. This results in enhancing the efficiency and reliability of the entire manufacturing scenario. COMSOL Multiphysics is a user-friendly software application that is used in engineering as well as science and academia. It offers two techniques for product design: virtual prototypes and multiphysics analysis. By utilizing these techniques, designers ensure the suitability and effectiveness of customized products. Altair HyperWorks is an effective CAE software tool developed by Altair Engineering. It helps designers perform various design iterations in customized manufacturing through modeling, simulation, analysis, and optimization. Altair Engineering provides these capabilities. MSC software provides a diverse range of simulation tools, including—but not limited to—MSC Nastran, Adams, and Marc, catering to various engineering disciplines and industrial sectors. Within the framework of personalized manufacturing, designers have the ability to conduct a multitude of simulations and analyses, encompassing kinematic, structural, and non-linear evaluations. Rhino3D, known as Rhinoceros 3D, is an impressive computer-aided design (CAD) program because of its powerful and adaptable free-form modeling features. Designers may create intricate parametric models using Grasshopper, a visual programming language and plugin for algorithm designs. The open-source CFD program OpenFOAM (Open Field Operation and Manipulation) has recently increased in popularity. Complex fluid flow and heat transfer problems can be simulated and analyzed using the software’s wide range of solvers and utilities.
2. Extension of the H-P2 layer.
  This layer, which includes the coordination of time constraints, resource allocation, and enhancements of those activities, is essential to the strategic planning of manufacturing procedures. Advanced planning and optimization technologies help production planners create and improve production schedules, which is how this is accomplished. These technologies make it easier to balance client requests, resource availability, and production capacity, satisfying the needs of individualized customization. Tools with higher levels of planning and optimization are shown in Figure 4b. A well-known enterprise resource planning (ERP) software company, SAP SE, provides the SAP Advanced Planning and Optimization (SAP APO) software solution. Planning for supply chains, production, demand, and elaborate scheduling are all aided by integrating multiple skills. The Oracle Supply Chain Management (SCM) software package includes the Oracle Advanced Supply Chain Planning (ASCP). It supports planners by providing a range of capabilities, including inventory optimization, demand planning, and supply chain planning, and eventually helps with customized personalization. IFS is a multinational company that offers a range of software solutions. IFS applications include comprehensive scheduling and planning capabilities, helping planners to enhance production efficiency by incorporating different constraints, such as customer demand, material availability, and capacity for customized goods and services. The Infor CloudSuite Industrial, also known as SyteLine, is developed by Infor. Infor is an internationally renowned enterprise that specializes in developing cloud-based enterprise software solutions. SyteLine is exclusively designed for the manufacturing industry. It helps planners to optimize the production planning scheduling of customized products by managing different factors, such as demand, capacity, and resource limitations. Kinaxis is a company that offers software for operation planning and supply chain management. The cloud-based supply chain management solution known as Kinaxis RapidResponse was created by this firm. The provision of end-to-end visibility across the supply chain enables enterprises to make better-informed decisions and respond more promptly to changes in demand or supply, especially in the context of customized personalization. Dassault Systèmes is responsible for the development of Quintiq, which is software utilized for supply chain planning and optimization. The purpose of this initiative is to aid enterprises in the effective administration and enhancement of their supply chain operations, logistics, and workforce scheduling. Demand Solutions DSX, a cloud-based program utilized for supply chain planning, was created by Demand Management, Inc. The system facilitates the optimization of production plans for customized products through the provision of demand planning, production planning, and inventory optimization capabilities, thereby enabling planners to enhance their operational efficiency. PLEX systems provide cloud-based enterprise resource planning (ERP) software to the manufacturing industry. Their flagship product, the Plex manufacturing cloud, connects and manages the manufacturing industry from the shop floor to the top floor. PlanetTogether is a company that provides advanced planning and scheduling (APS) software. It is designed to help manufacturing industries optimize their scheduling processes and production planning, improve resource utilization, enhance overall operational efficiency, and reduce lead time. LLamasoft Supply Chain Guru, developed by the Llamasoft company, is software for supply chain modeling and optimization. Industries utilize it to model, simulate, and optimize their supply chain operations. By leveraging these advanced planning and optimization tools, planners can create efficient production plans that cater to the personalized customization needs of new model factories.
3. Extension of the H-P3 layer.
  Advanced inventory management tools help industries monitor real-time inventory levels and automate replenishment processes to meet personalized customization. These tools are shown in Figure 5a. Fishbowl Inventory is a popular inventory management software solution primarily designed for small- and medium-sized enterprises. It integrates with QuickBooks, providing real-time tracking of inventory levels, advanced reporting, and automated reordering for better decision-making in personalized manufacturing. Oracle NetSuite is a comprehensive cloud-based business management software that includes financial management, order management, revenue management, and inventory management. System Applications and Products (SAP) in data processing is a German multinational software corporation that makes enterprise software to manage business operations and customer relations. It deals with the management of stocks, either by value or quantity, and oversees planning, entry, and documentation of all goods movements within the warehouse, to, and from it. Zoho Inventory is a cloud-based inventory management (CBIM) software designed for small- to-medium-sized enterprises. It is part of the Zoho suite of applications, which includes tools for project management, CRM, HR, and more. It helps to create and manage both sales and purchase orders and track inventory, which leads to managing the order fulfillment processes. Cin7 is a CBIM software that is designed to keep track of inventories across multiple channels and locations. It encompasses different functionalities, such as warehouse management, inventory management, and point of sale. DEAR Inventory is a renowned CBIM software that is specifically designed for small- to medium-sized enterprises. It encompasses different business software solutions for seamless operations. It helps industries to streamline and automate inventory and order management. Unleashed Software has significant importance in small- and medium-sized enterprises. It is a CBIM software that aids in managing inventory tasks. It offers a range of functionalities and features to help industries efficiently manage their streamlined operations and inventory, as well as improve overall efficiency. Infor CloudSuite Industrial is an enterprise resource planning software designed for manufacturing industries, helping them track inventory levels in real time, optimize processes, streamline operations, and improve overall performance. Odoo Inventory is a module within the Odoo ERP system that focuses on inventory management and control. It is a comprehensive inventory management software designed to help industries efficiently manage their stock levels, optimize inventory operations, streamline warehouse operations, and track inventory movements. TradeGecko is a cloud-based inventory and order management software designed for small- and medium-sized enterprises. It offers a range of functionalities and features to help industries efficiently manage their streamlined operations and inventory, as well as enhance overall efficiency.
4. Extension of the H-P4 layer.
  Advanced logistics management tools, such as real-time tracking systems and route optimization tools, enable industries to efficiently and cost-effectively deliver products in newly personalized customization factories. Some of these tools are shown in Figure 5b. Oracle Transportation Management (OTM) is a comprehensive transportation management software solution offered by the Oracle Corporation. It assists both shippers and logistics service providers by providing a single platform for planning, freight payment, execution, and business process automation. SAP Transportation Management (SAP TM) is a comprehensive transportation management software solution offered by SAP. This software assists various industries in strategizing, enhancing, and implementing their transportation operations. This software helps industries increase their customer service, minimize costs, and streamline logistics procedures. BluJay Solutions is an enterprise that specializes in offering software solutions for supply chain management and transportation management. This company’s solutions are designed to enhance transportation and logistics management, boost organizational efficiency through supply chain optimization, improve customer service, and enhance customer care. The Descartes Systems Group is a well-known supplier of software systems for controlling supply chains and logistics worldwide. The company provides a wide range of goods to improve the efficiency and visibility of business logistics operations. The Manhattan Associates transportation management system (TMS) is primarily recognized as the best option for managing supply chains and Omnichannel commerce. To assist businesses in enhancing customer service, streamlining transportation operations, reducing costs, and enabling more efficient deliveries, the transportation management system (TMS) was developed. The C.H. Robinson Transportation Management Center (TMC) is an international business that specializes in third-party logistics (3PL), offering logistical and transportation-related services. It aims to maximize the efficiency of transportation operations while enhancing the efficiency of supply chain operations. This company’s use of contemporary technology, its dependence on the knowledge of seasoned industry professionals, and maintenance of an extensive supplier network enable it to provide complete transportation management services, from planning to execution. The market acknowledges MercuryGate TMS as a premier provider of TMS software. Businesses may make the most of their logistics and transportation operations and improve the efficiency of their supply chain as a whole with the help of this service. JDA TMS is a notable software-based solution that simplifies the transportation and logistics tasks in supply chains. Making the methods for moving things as efficient as possible can help businesses be more productive, save money, and provide their customers with better service. By using Paragon Routing and Scheduling software, logistics operations can automate and make the process of routing and arranging vehicles much more efficient. This is possible because the software works with systems from different parts of the world. With this service, businesses can receive assistance with organizing and managing their transportation and service routes. They can use their resources more wisely and save money as a result. Route4Me allows users to access software in the cloud, which is accessible online. It was made to make it easier for users to find the best routes and run fleets of vehicles. It allows for a wide range of business plan delivery routes, allows users to make changes to current routes, and track the progress of deliveries. This makes it possible to ship personalized things in a way that is fast and cheap. With Route4Me, businesses can easily plan, handle, and control how their delivery teams are spread out. This provides them with a big edge over their competitors.
5. Extension of the H-P5 layer.
  In this layer, the systems contribute to a newly personalized customization factory, along with the mechanism, as shown in Figure 6a. In this figure, supply chain integration connects suppliers and customers in real time, resulting in optimizing production planning, inventory management, and logistics. This integration ensures a more responsive and agile supply chain, which is capable of adapting to fluctuations in demand and minimizing lead times. Human–machine integration ensures seamless collaboration between human workers and machines, which leads to more adaptable and efficient production processes. Human workers provide problem-solving, creativity, and oversight, while machines handle heavy lifting and repetitive tasks. A flexible manufacturing system (FMS) allows for the quick and efficient reconfiguration of production lines to accommodate variations in product specifications. An FMS enables greater adaptability and customization in response to customer needs. The mechanism of personalized customization for a new model factory contains the following steps.
  - Customer data integration: Customers share their requirements or preferences, such as materials, colors, sizes, or any other entity, through various sources. These channels may include online interfaces, configurators, CRM (customer relationship management) systems, or direct contact with the manufacturer. After analyzing data, digital product models are generated.
  - Product configuration: After collecting customer data, the newly personalized customization factory offers product configurations based on individual needs. Customization options are guided by the firm’s production capabilities and constraints; customers can customize their required products by choosing different options, like the size, color, feature, and material.
  - Real-time data exchange: The newly personalized customization factory integrates and exchanges data from production systems, customer orders, and supply chain partners to facilitate seamless communication throughout the manufacturing ecosystem. Manufacturers can obtain different information, like production status, resource allocation, and material availability by connecting various production stages.
  - Quality control and testing: Quality parameters can be tracked through sensors and monitoring systems to identify flaws or deviations. In this way, quality control measures are deployed throughout manufacturing to ensure that the personalized products meet the desired quality standards and customer requirements.
  - Customer engagement, traceability, and feedback: The newly personalized customization factory allows manufacturers to engage customers by offering delivery updates and personalized notifications. This factory also offers technologies, like barcodes and RFID tags, to trace the customized products throughout the manufacturing process to enable customers with real-time updates and transparency. Even after the sale, these interactive interfaces for customization help introduce new features or functionalities based on customer feedback.
6. Extension of the H-P6 layer.
  Advanced technologies contribute significantly to newly personalized customization factories. Some of these technologies will be discussed, as shown in Figure 6b. Artificial intelligence (AI) and machine learning (ML) enable the system to analyze a vast amount of data from the factory floor, optimize processes, identify patterns, trends, and correlations, enable greater customization of products, and maintain high levels of quality and efficiency. Additive manufacturing, also known as 3D printing, helps to produce complex geometries with minimal waste and setup times. It is well-suited for the production of personalized products, as it can create unique products without the need for expensive molds or tooling. Robotics and automation deploy industrial robots and automated systems to perform repetitive tasks with high speed and precision, reducing labor tasks and minimizing errors. IoT and sensor networks help to gather and transmit real-time data from the factory floor, facilitating predictive maintenance, informing decision-making, and improving operational efficiency. Digital twin creates a virtual replica of the manufacturing process for real-time monitoring, analysis, and optimization. It helps to identify bottlenecks, predict equipment failures, and enhance efficiency by optimizing production parameters. Data analytics interprets and processes data from different sources, enabling data-driven decision-making. Visualization tools help decision-makers to better understand complex data and make informed choices accordingly. Cybersecurity has significant importance in the newly personalized customization factory. As the new factory relies heavily on data exchange and interconnected systems, a robust cybersecurity strategy is mandatory to protect sensitive and confidential information and maintain the integrity of the production process. Moreover, the security of different domains is essential to extend the framework as discussed below.
  (a) Physical security: The physical security of an IoT-enabled manufacturing system contains the following aspects.
    - Device hardening: The devices within the manufacturing system need to be physically robust, tamper-proof, and secure enclosures. It will prevent the system from intentional physical attacks and protect against environmental factors, such as moisture, dust, or temperature changes.
    - Physical access control: Robust access control mechanism is necessary to secure manufacturing facility access. It may contain biometric identification systems, key cards, access codes, and security personnel to limit access to authorized personnel only. In addition, data servers and sensitive equipment should be kept in safe and secure locations.
    - Device location and tracking: To prevent unauthorized removal, it is essential to have the physical locations of all devices within the manufacturing ecosystem. For that purpose, devices are engaged with GPSs or other tracking technologies to permit monitoring and tracking at any time.
    - Proper disposal and decommissioning: Devices that are not in use must be disposed of securely to avoid data leakage.
    - Monitoring and surveillance tools: Installing monitoring and surveillance tools, such as CCTV cameras, motion detectors, and intrusion detection sensors, enable the system to detect tampering with IoT devices and unauthorized access to the manufacturing facility.
    - Redundancy and resilience: A contingency plan must be available due to any physical attack to back up the critical system and data.
  (b) Application security: The application security of an IoT-enabled manufacturing system possesses the following aspects.
    - Secure coding practices: Secure coding practices must be implemented during application security development. These practices may include input validation, coding standards, proper error handling, and least privilege principles to lessen the effects of vulnerabilities caused by attackers.
    - Security testing and code reviews: Regular and comprehensive testing and code reviews must be conducted to identify security flaws, vulnerabilities, or design weaknesses in the applications. This can be done using techniques like static application security testing (SAST), dynamic application security testing (DAST), manual code reviews, and third-party security assessments.
    - Authentication and authorization: Robust authentication mechanisms should be implemented to authenticate users and devices and to ensure that only legitimate users can access the features or data. It may include techniques like two-factor authentication, username/password authentication, digital certificates, and role-based access control.
    - Secure deployment and maintenance practices: Before putting them into production, secure deployment practices should be implemented to ensure that the applications are correctly hardened and configured. The process of regular updating helps to secure from malicious updates.
    - Application programming interface (API) security: IoT-enabled manufacturing systems depend heavily on APIs for communication between devices and applications. It is vital to secure APIs; the security techniques involved include input validation, rate limiting, and secure API gateways.
  (c) Device security: Device security is a critical aspect that ensures the system’s overall security; it has the following key aspects.
    - Hardware security: IoT devices must be designed with hardware that enables security features. It may include crypto accelerators, tamper-proofing, secure boot mechanisms, and hardware random number generators.
    - Device authentication: Before communication starts, to join the network, the devices must authenticate and have unique identities. This may involve certificates or other forms of robust authentication.
    - Firmware and software integrity: The integrity of device firmware and software must be ensured to avoid unauthorized access or tampering. It may involve secure boot mechanisms, digital signatures, or checksums to validate the authenticity and integrity of firmware updates. Firmware updates must be appropriately signed and encrypted to avoid installing malicious software.
    - Least privilege principle: Careful considerations are required while using the devices and their applications. These must operate using the least privilege principle, depending on the functions, to prevent the security breach’s potential damage.
    - Anomaly detection: Devices should be monitored regularly to prevent security issues, such as unexpected traffic flow or changes in power usage.
  The security of IoT-enabled manufacturing systems is necessary due to their interconnectedness and potential vulnerabilities to provide the integrity of industrial operations and ensure the safety of the manufacturing ecosystem. In order to guarantee the security of the reorganized system due to customized operations, the following security measures must be implemented.
  - Data encryption: The confidential data should be encrypted during transmission to avoid tampering or unauthorized access.
  - Access control: Role-based access control mechanisms and identity management protocols are introduced to only provide authorized personnel access. By doing so, only authorized personnel can interact with or modify the system.
  - Regular audits: To identify and tackle vulnerabilities in the system, regular audits are mandatory.
  - Device authentication: Before communicating within the network, IoT devices must be authenticated to prevent unauthorized devices from intervening. Robust authentication mechanisms are used for this purpose.
  - Intrusion detection and monitoring: Any unusual or malicious activities within the manufacturing system can be detected by real-time monitoring tools and intrusion detection systems. This will mitigate security issues and promote timely responses.
  - Security updates and patches: The manufacturing system containing IoT devices and sensors must be regularly updated and securely configured with patches to fix any security loopholes and protect against the latest threats.

#### 3.1.2. Framework for a Network-based Manufacturing System

Wired and wireless communication technologies link and transfer information across equipment, enterprises, and control systems to achieve interconnection and interoperability. The interconnection and compatibility layer of smart characteristics is selected from SMS. This layer is further extended to a smart-interconnected factory, including in the SMIS with the IoT-enabled NBMS selected as a system model. This NBMS is further secured from the black hole attack using the NTRUEncrypt algorithm. The proposed framework is shown in Figure 2.

### 3.2. System Model

**Figure 7:**
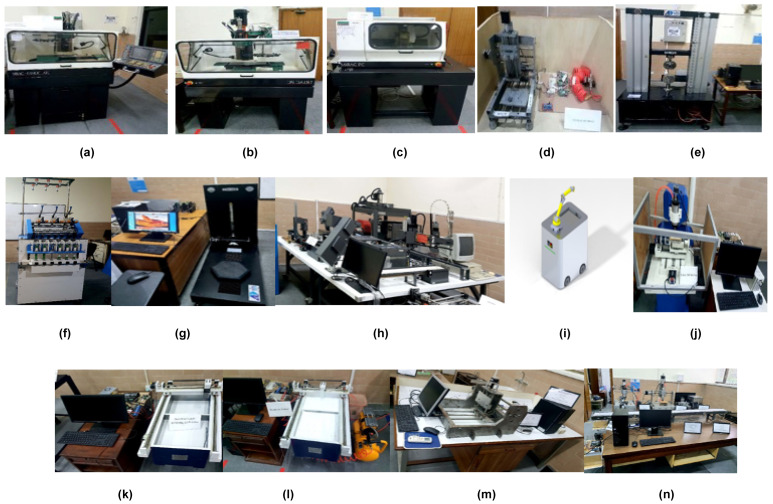
(**a**) CNC milling 1, (**b**) CNC milling 2, (**c**) CNC turning, (**d**) Pneumatic jet printer, (**e**) universal tensile testing machine, (**f**) spinning machine, (**g**) 3D laser scanner, (**h**) retrofitted FMC, (**i**) mobile industrial robot (MiRs), (**j**) 5-axis CNC milling machine, (**k**) selective laser sintering 3D printer, (**l**) binder jet printer, (**m**) precision router, and (**n**) indigenous FMC.

The network-based manufacturing system comprises thirteen workstations with eleven single machine cells and two flexible manufacturing cells (FMC), with one acting as the master controller. It also contains seven mobile industrial robots (MiRs), one for each pair of workstations, and the last one for the retrofitted FMC acting as the master controller. The NBMS under consideration has CNC machines, a material handling and storage system, and a loading and unloading station. All machines are placed at different workstations and can perform subtractive and additive manufacturing operations, respectively. MiRs enable the material transportation facility, reducing labor and production bottlenecks. All the machines with their names are shown in Figure 7.

### 3.3. Network Model

**Figure 8:**
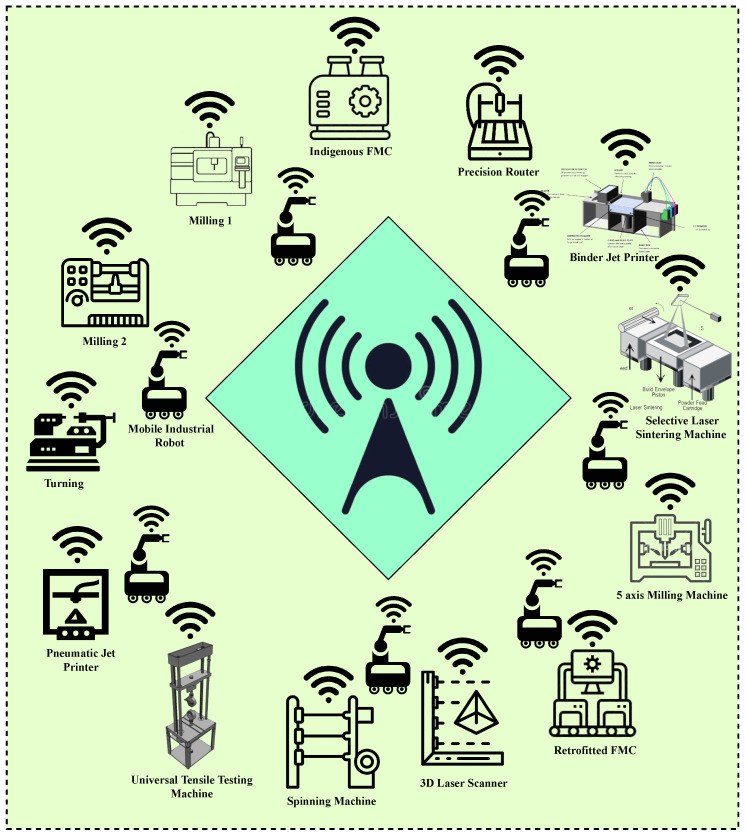
Network model of the NBMS.

The network model of NBMS consists of 20 nodes, representing 11 sensor-enabled single machine cells, 2 sensor-enabled flexible manufacturing cells, and 7 sensor-enabled mobile industrial robots. All the nodes are IoT-enabled and capable of performing functions remotely. The CNC machines can also communicate through the access point, as shown in Figure 8.

### 3.4. Effect of the Black Hole Attack in the IoT-Enabled AODV Network

The AODV protocol was developed primarily for mobile ad hoc networks and is regarded as a flat-based routing mechanism. It can also be used for IoT-enabled mobile nodes. The AODV protocol is based on the distance vector method. It may be viewed as a development of the proactive DSDV method, which stands for “Destination-Sequenced Distance-Vector Routing”. AODV is ad hoc on-demand routing protocol, which decreases the number of message broadcasts compared to DSDV. AODV adopts two procedures: “RD” for route discovery and “RM” for route maintenance. The AODV protocol defines three well-known messages, called route request (RREQ), route reply (RREP), and route error (RERR). During the crucial “Route Discovery” step of the AODV, “RREQ” messages are consistently sent out over the IoT-enabled network. When the respective node obtained an “RREQ” message, it immediately sent a reverse route “RREP” message. “RERR” messages are sent to the destination and source nodes immediately when the routes are broken. AODV operates well in low-density networks and mobile nodes [47].

**Figure 9:**
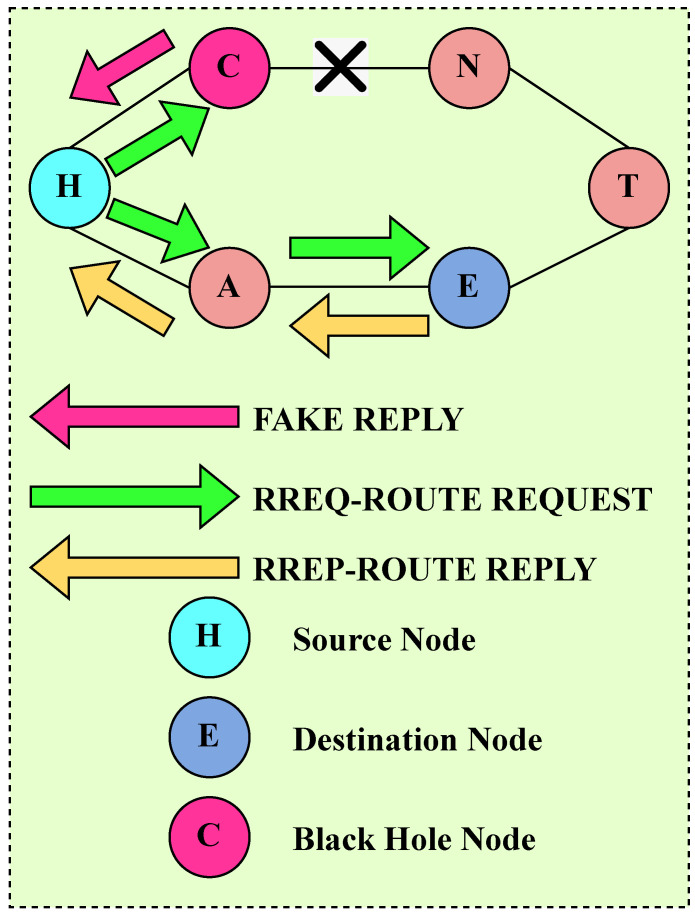
The data packet destruction due to the black hole attack.

The black hole attack (BHA) lies in the category of the network layer attack. The BHA node drops all the data and affects the overall network traffic [48]. In the BHA, the malicious node waits for the RREQ message to come from the neighboring nodes. After receiving the RREQ messages, it immediately sends the fake copy of RREP messages with the maximum sequence number before the actual response of other nodes. A malicious node pretends one of the nodes has a suitable path to the destination. By doing so, the sender node acknowledges that the route discovery has been acquired and starts sending data to the malicious node. In this way, the malicious node drops all the data, which affects the network traffic badly. This phenomenon is shown in Figure 9, “H” is the sender node, “C” is the BHA node, and “E” is the destination node.

### 3.5. NTRUEncrypt Cryptography

Conventional algorithms, such as RSA and ECC, are not appropriate for the IoT environment; the reason lies in the complexities of encryption and decryption operations. Compared to these algorithms, NTRUEncrypt is efficient in terms of speed, detection accuracy, and security level [49]. In NTRU, polynomial executions in the ring are simple, making it faster. The “*N*th-Degree truncated polynomial ring unit” (NTRU) is a lattice-based public key cryptographic technique that uses the algebraic structure of the polynomial ring “R” [50]. “R” is the ring of convolution or (truncated) polynomials with degree “N−1”, with integer coefficients in the form of $a_{0}+a_{1}X+a_{2}X^{2}+\ldots +a_{N-2}X^{N-2}+a_{N-1}X^{N-1}$. Here, $a_{N}$ denotes coefficients of the polynomial, depending on the NTRUEncrypt variant being used. The coefficients are usually binary (0 or 1) or trinary (−1, 0, or 1). *X* represents the variable of the polynomial.

“R” can be defined as [50]:

(1) $$R=\left(\frac{Z[X]}{\left(X^{N}-1\right)}\right).$$

With the modulo “*p*” and “*q*”, the ring “R” can be written as

(2) $$R_{p}=\frac{\left(Z/pZ)[X\right]}{\left(X^{N}-1\right)}\;\mathrm{and}\;R_{q}=\frac{\left(Z/qZ)[X\right]}{\left(X^{N}-1\right)}.$$

The following parameters characterize the implementation of NTRUEncrypt:

- *N*: Degree Parameter.
- $D_{f},D_{g}$: Spaces for private keys (private keys are chosen from these sets of small polynomials).
- p,q: Small and large moduli.
- $D_{m}$: Space for plaintext (set of polynomials representing encryptable messages).
- $D_{r}$: Space for the blinding value (the temporary blinding value used for encryption from this set of polynomials).
- Center: Centering method (performs mod ’*q*’ reduction during decryption).

Key creation:

i. Generate polynomials *g* and *f* randomly in $D_{g}$ and $D_{f}$.
ii. Take the inverse of *f* in $R_{p}$ to obtain $f_{p}$, and the inverse of *f* in $R_{q}$ to obtain $f_{q}$, making sure that *g* is invertible in $R_{q}$.
iii. Create $\left(f,f_{p}\right)$ as the private key and $h=p*g*f_{q}\;\left(\mathrm{mod}\;q\right)$ as the public key.

Encryption:

i. Choose a small polynomial randomly, such that $r\in D_{r}$.
ii. Compute e=r∗h+m(modq), called ciphertext.

Decryption:

i. Compute a=center(f∗e), where the center decreases its input into the interval [B,B+q−1].
ii. Retrieve *m* by computing $m=f_{p}*a\;\left(\mathrm{mod}\;p\right)$, where a=p∗r∗g+f∗m(modq), using $h=p*g*f_{q}$ and e=r∗h+m. Hence, after suitable choices of parameters and the centering operation, the term p∗r∗g disappears and $f_{p}*f*m=m\;\left(\mathrm{mod}\;p\right)$.

NTRUEncrypt cryptography helps provide the secure encryption and decryption of data. It can resist different types of attacks, including cryptanalytic attacks, factorization attacks, brute-force attacks, chosen-ciphertext attacks, quantum computing attacks, and timing attacks [50]. Conversely, a combination of NTRUEncrypt cryptography and the AODV routing protocol can tackle various attacks, such as DoS attacks, rushing attacks, routing table overflow attacks, false data injection attacks, black hole attacks, wormhole attacks, Sybil attacks, man-in-the-middle attacks, spoofing attacks, replay attacks, key management attacks, and side-channel attacks.

### 3.6. System Setup

**Figure 10:**
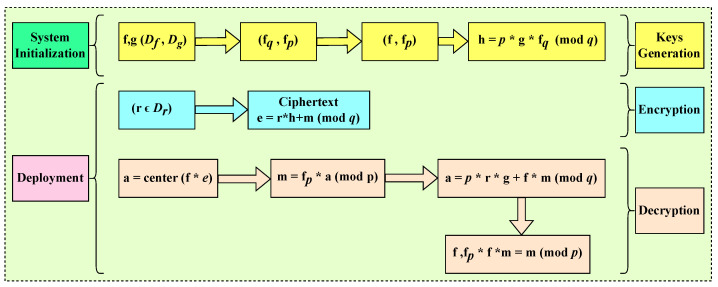
NBMS initialization and deployment.

The system setup consists of two main parts: system initialization and deployment. In the system initialization phase, all the necessary parameters, such as the private key and public key, are generated and stored in each machine. This involves implementing a key generation mechanism to generate the required cryptographic keys. In the deployment phase, encryption and decryption mechanisms are employed to ensure secure communication within the network-based manufacturing system. The process of system initialization and deployment is illustrated in Figure 10.

**Figure 11:**
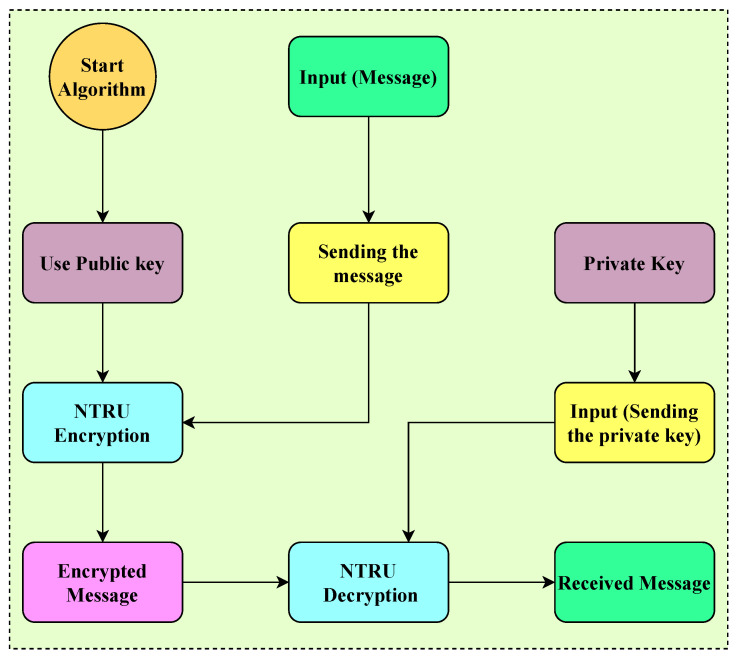
NTRUEncrypt encryption and decryption between the sender and receiver.

The NTRUEncrypt encryption and decryption mechanism is illustrated in Figure 11. In this mechanism, the public key of any manufacturing cell node in the NBMS is known to the other nodes, while the private key is kept confidential. The encrypted message, also known as the ciphertext, comprises the receiver’s public key encrypted with the sender’s message. The decrypted message, referred to as the plaintext, is obtained by decrypting the ciphertext using the private key.

#### NTRUEncrypt-Secured IoT-Enabled NBMS

**Figure 12:**
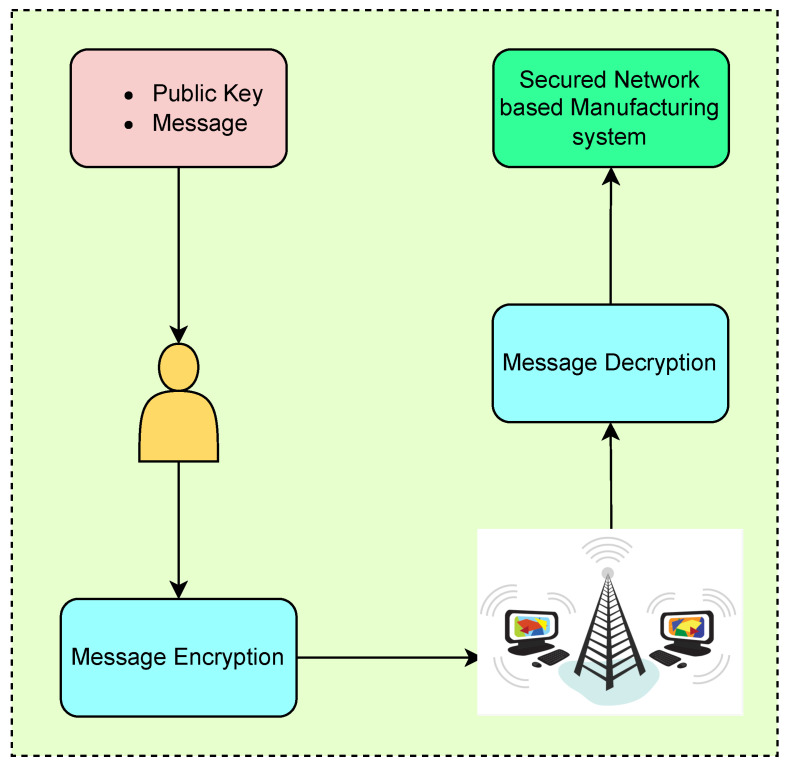
Secured IoT-enabled NBMS.

Figure 12 illustrates the secure communication process between the user and the respective machine in the IoT-enabled network-based manufacturing system. The user of one machine sends an encrypted message to another machine through the internet, and the receiver machine decrypts the message to obtain the original plaintext. This process ensures secure communication using the NTRUEncrypt encryption and decryption mechanism, as depicted in Figure 11.

### 3.7. Computational Complexity

Table 2 describes the computation complexity of the AODV routing protocol for each route discovery and route maintenance phase, where *n* is the number of nodes in the network and involves the linear-time operations O(n) for route discovery and route maintenance. Hence, the complexity of the AODV protocol is lightweight. Similarly, Table 3 describes the complexity of the NTRUEncrypt cryptography technique. Here, η shows the security parameter determining the sizes of keys. It can be observed from the table that the cryptography technique increases the computational complexity of the algorithm. Nevertheless, O(ηlogη) is log-linear complex, which does not degrade the performance of the proposed system [51].

### 3.8. Simulation Setup

The proposed scheme uses NS-2.35 for simulating the network model of NBMS. The initial setting of all the networks is considered the same to fairly evaluate the performances of all the considered networks. The simulation parameters are shown in Table 4.

### 3.9. Experimental Model

**Figure 13:**
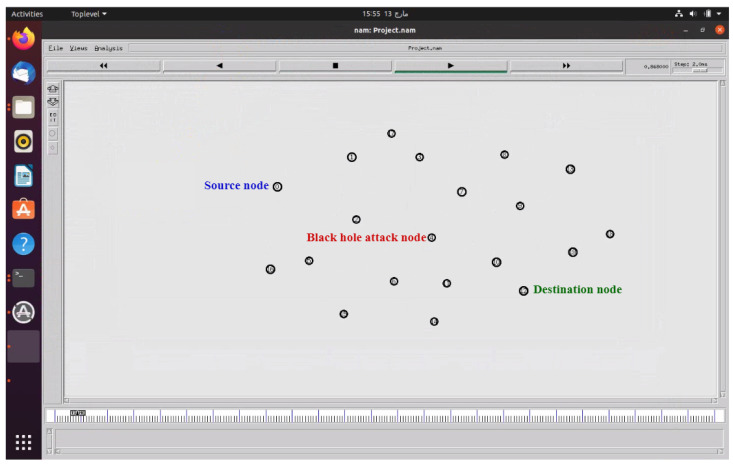
Simulation setup.

The experimental model consists of 20 wireless nodes represented by circles. These nodes form an IoT-enabled network-based manufacturing system. Node “0” is designated as the source node, while node “12” serves as the destination node. Node “4” is configured as the BHA node, as depicted in Figure 13. All nodes in the system are mobile nodes. The system uses the network animation (NAM) tool of NS2 to visualize the movement of nodes [52]. As shown in Table 4, it uses the AODV protocol to simulate the considered networks [32]. Moreover, the two-ray ground wireless propagation model is used, which considers the line-of-sight as well as the reflected signals during simulation [53]. Considering the random-way point model, a simulation area of 1100 × 1100 m${}^{2}$ is used. Additionally, it employs the NTRUEncrypt technique to address the BHA threat. The system conducts three independent simulation scenarios, including a healthy network simulation, a black hole attack network simulation, and an NTRUEncrypt-secured network simulation. It generates the results from the trace file of NS2 via AWK scripts.

## 4. Evaluation Metrics

### 4.1. Throughput (TP)

TP is a key performance metric in networking that measures the rate at which data packets are successfully transferred between the source and destination. Instantaneous throughput is defined as the sum of all the packets received during a specific time interval. The average throughput, on the other hand, considers the data packets received within a unit of time at the receiver node [54]. The network’s throughput can be calculated using the following equation:

(3) $$TP=\frac{\sum S_{RP}}{\sum \Delta t},$$

where $S_{RP}$ represents the sizes of received packets, and Δt is the time duration between the stop time $F_{t}$ and the start time $E_{t}$.

### 4.2. Goodput

Goodput is a metric that quantifies the rate at which valuable data are successfully transferred over the network and reach their intended destination, ensuring data integrity. Instantaneous goodput is calculated for a specific time interval [55]. The formula for goodput is given by

(4) $$Goodput=\frac{\mathrm{Size}\;\mathrm{of}\;\mathrm{the}\;\mathrm{transmitted}\;\mathrm{file}}{\mathrm{Time}\;\mathrm{taken}\;\mathrm{to}\;\mathrm{transfer}\;\mathrm{the}\;\mathrm{file}.}$$

Goodput focuses on relevant and meaningful data, excluding data retransmission, protocol overheads, or any other extraneous information. It also excludes the packets that were delivered but, later on, discarded because they were corrupted or not delivered on time to their intended destination. In the context of IoT-enabled NBMS, goodput refers to the useful data rate that is actually processed, acted upon, and contributes directly to the performance, functionality, efficiency, and security of the system [56]. Hence, goodput is a measure of the network’s deliverable capacity for useful data.

### 4.3. E2E Delay

The time needed for a packet to travel from the source to the destination across a network is known as the E2E delay or one-way delay [57]. Its formula is given as follows:

5. $$E2E=\frac{\sum A_{t}\cdot S_{t}}{\sum N_{L}},$$

where $A_{t}$ represents the arrival time of a packet, $S_{t}$ represents the sent time of a packet, and $N_{L}$ represents the number of links traversed by each packet.

### 4.4. Packet Delivery Ratio

The PDR is calculated as the total number of received packets divided by the total number of packets sent from sources, expressed as a percentage [58]. Its formula is given as follows:

6. $$PDR=\frac{\sum P_{R}}{\sum P_{S}}\times 100.$$

where $P_{R}$ represents the total number of received packets and $P_{S}$ represents the total number of packets sent.

## 5. Results and Discussion

This section presents the simulated results for healthy, malicious, and NTRUEncrypt-secured networks under various evaluation metrics, such as instantaneous throughput, instantaneous goodput, average end-to-end delay, average throughput, and packet delivery ratio.

### 5.1. Instantaneous Throughput

#### 5.1.1. Healthy Network

**Figure 14:**
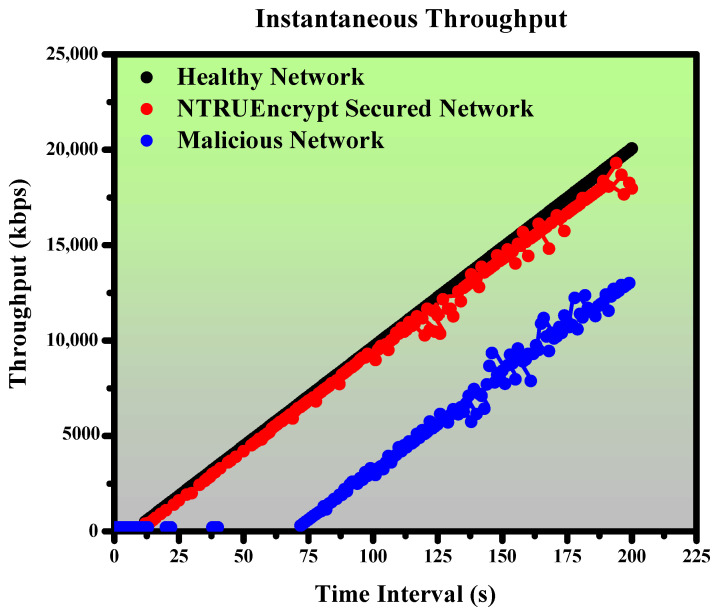
Instantaneous throughput for the healthy network, malicious network, and the NTRUEncrypt-secured network.

A healthy network is taken to check the trend of instantaneous throughput. Figure 14 shows that instant throughput increases linearly; the value starts to grow from 10 s because of the time spent generating the packets before that value. After generating packets, the graph shows the linear relation and jumps to a maximum value of about 20,000 kbps at 200 s.

#### 5.1.2. Malicious Network

In this case, the malicious node (black hole-attack node) attacks the healthy network to check the system’s behavior. Node 4 is an attacking node that is used to make the black hole attack network. Figure 14 shows that throughput did not even increase up to 72 s. The reason is that the malicious node pretends to be an efficient relay, attracts more packets, and then immediately drops those packets instead of forwarding them to the destination. Moreover, it creates network congestion, as other nodes continuously attempt to resend the lost packets that lead to bottlenecks and cause a reduction in throughput. Additionally, the malicious node misdirects the incoming traffic into an alternative or longer path that does not lead to the desired destination, causing delays and latency in data transmission and decreasing the throughput. However, the trend changes abruptly after 72 s; the graph shows the increase in throughput; the reason behind this is rerouting. The system finds the optimal path where the malicious node is unavailable. The malicious network under the black hole-attack node gives a throughput of 13,000 kbps at 200 s because the attacking node drops many packets during routing, unlike a healthy network, where that value reaches 20,000 kbps.

#### 5.1.3. NTRUEncrypt-Secured Network

The malicious network is protected by NTRUEncrypt-secured routing in this case. The NTRUEncrypt-secured network checks the system’s behavior with a secured algorithm in the presence of the malicious node. Figure 14 shows the increase in throughput even under the attacking node through the entire time frame. The network achieves 19,300 kbps at 200 s, which shows that our system remains secure under an attacking node. The slight decrease in value, compared to the healthy network, is due to the cryptographic overhead of the NTRUEncrypt algorithm.

### 5.2. Instantaneous Goodput

#### 5.2.1. Healthy Network

**Figure 15:**
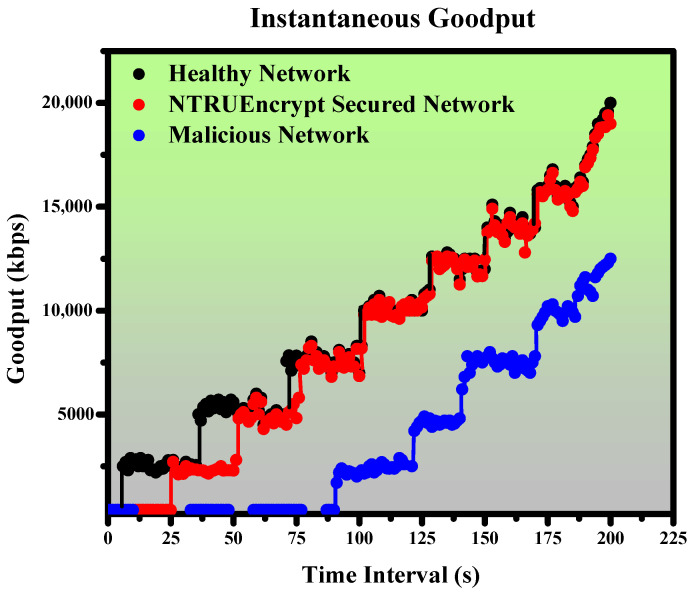
Instantaneous goodput for the healthy network, malicious network, and NTRUEncrypt-secured network.

Figure 15 shows that goodput increases with a notable trend after every 20–30 s. The system takes the first 9 s to generate the packets, and after that, the system finds the rerouting and shows a rapid increase due to rerouting. Hence, the value moves up to 2500 kbps at 9 s. Later, the system finds the optimal path due to rerouting every 20 to 30 s and shows the increase in goodput up to 20,000 kbps at 200 s.

#### 5.2.2. Malicious Network

The malicious network shows a different trend compared to the healthy one, as depicted in Figure 15. In the initial ninety seconds, the system does not show any increase in goodput value due to the attacking node, and the system is under recovery. After 90 s, the system shows a sudden increase in goodput value due to rerouting, and the network finds the optimal path to send the packets from the source to the sink node. The value only moves up to 12,500 kbps at 20 s due to the interference of the malicious node.

#### 5.2.3. NTRUEncrypt-Secured Network

Figure 15 shows that goodput increases with a notable trend every 25 s. The system takes the first 25 s to generate the keys, and after that, the system finds the rerouting and shows a rapid increase due to rerouting. Hence, the value goes to 2500 kbps at 26 s. Later, the system finds the optimal path due to rerouting almost every 25 s and shows the increase in goodput, up to 19,500 kbps at 200 s. It shows that our system operates safely under a black hole attack.

### 5.3. Average E2E Delay

**Figure 16:**
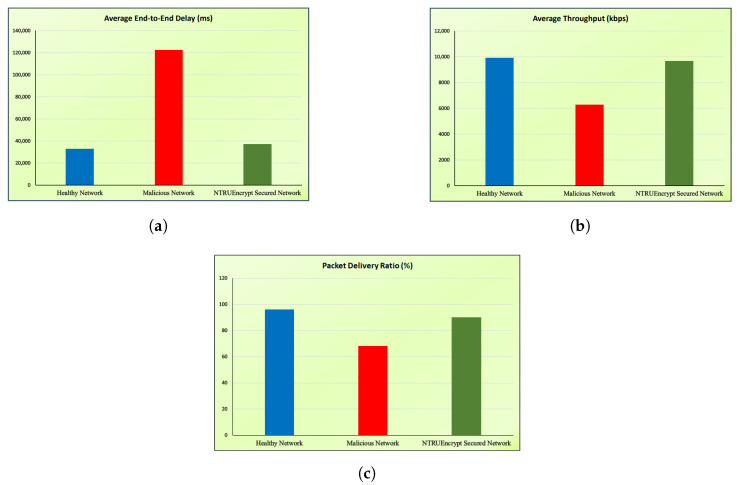
(**a**) Average E2E delay for healthy, malicious, and NTRUEncrypt-secured networks; (**b**) average throughput for healthy, malicious, and NTRUEncrypt-secured networks, and (**c**) PDR for healthy, malicious, and NTRUEncrypt-secured networks.

Figure 16a shows that the average E2E delay for a malicious network is much higher than the NTRUEncrypt-secured and healthy network of 20 nodes. This is because packet drops increase in the malicious network because an attacking node is added to this network without any precautionary measures, and data take time to reach the sink/destination node. The other reason is the node’s unavailability, so the source node selects another alternative path. By doing so, time consumption occurs, and it causes delays. The average E2E delay of the NTRUEncrypt-secured network slightly increases compared to the healthy network because of the associated overhead of the cryptographic algorithm. However, NTRUEncrypt achieves significant improvement when compared to the malicious network, resulting in a decrease in delay in the presence of an attacking node.

### 5.4. Average Throughput

Figure 16b shows that the average throughput of the malicious network is less than that of a healthy and NTRUEncrypt-secured network of 20 nodes. This is because the attacking node hinders the network from sending the complete packets as the same vogue observed in the case of instantaneous throughput. With the NTRUEncrypt-secured network, the average throughput offers a reasonably efficient value that is not far from the healthy network. It shows that the system behaves safely even in the presence of the black hole-attack node.

### 5.5. Packet Delivery Ratio

Figure 16c shows that the PDR of the malicious network is less than the healthy and NTRUEncrypt-secured network of 20 nodes. This is because the packets received are less; the network drops many packets during the routing due to an attacking node. In addition, network congestion occurs due to packet loss, hindering the proper packet delivery. In the NTRUEncrypt-secured case, the performance is not far behind the healthy one because of the decrease in packet drop value.

### 5.6. Performance Comparison under Different Workloads

This subsection presents performance comparisons of the considered networks under different workloads for E2E delay, average throughput, and PDR.

#### 5.6.1. Average E2E Delay

**Figure 17:**
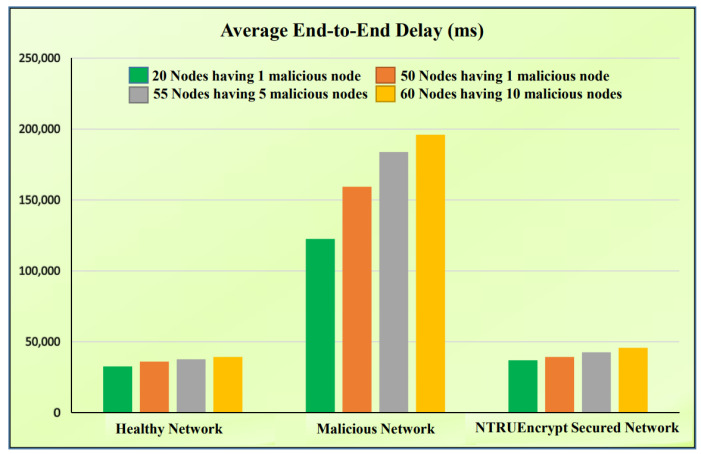
Average end-to-end delay for different node numbers.

Figure 17 shows the E2E delay for different numbers of normal and malicious nodes, as discussed below.

1. A total of 50 normal nodes and 1 malicious node.
  - Healthy network: Figure 17 shows that the E2E delay in the 50-node scenario is higher than the 20-node scenario. This is due to the addition of nodes leading to potentially longer paths and increased contention.
  - Malicious network: The delay in the 50-node scenario increases when compared to the 20-node case because an increasing number of nodes can amplify the disruption effect of the malicious node, and the malicious node in a more extensive network impacts more routes. It results in increased delay due to rerouting.
  - The NTRUEncrypt-secured network: The delay in the 50-node scenario increases compared to the 20-node scenario because the protective mechanism prevents rerouting delays caused by a malicious node, but the cryptographic overhead from NTRUEncrypt could introduce some additional delay.
2. A total of 50 normal nodes and 5 malicious nodes.
  - Healthy network: Figure 17 shows that the E2E delay in the 50-node scenario is higher than the 20-node scenario because increasing the number of nodes means more hop counts for a packet to traverse, resulting in more delay.
  - Malicious network: In high-density scenarios, the delay increases compared to the 20-node case. With five black hole-attacking nodes, there is a higher chance for routes to be compromised, causing packets to reroute and subsequently leading to higher delays.
  - The NTRUEncrypt-secured network: The 50-node delay increases compared to the 20-node scenario because of the cryptographic processing overhead from NTRUEncrypt; the need for potentially frequent route discoveries due to the presence of black hole nodes can increase delay, even if the protection mechanism avoids some rerouting.
3. A total of 50 normal nodes and 10 malicious nodes.
  - Healthy network: Figure 17 shows that the E2E delay in the 50-node network is higher compared to the 20-node scenario because the denser network can result in longer paths and more chances of communication congestion, leading to higher E2E delay.
  - Malicious network: A higher-density delay is potentially much higher than in the 20-node case. This is because the ten black hole nodes will disrupt the network, causing frequent rerouting and more extended path discoveries. It also means higher chances of packets encountering malicious routes, leading to a substantial delay.
  - In the NTRUEncrypt-secured network, the delay increases in high density compared to the 20-node setup due to the cryptographic overhead from NTRUEncrypt.

#### 5.6.2. Average Throughput

**Figure 18:**
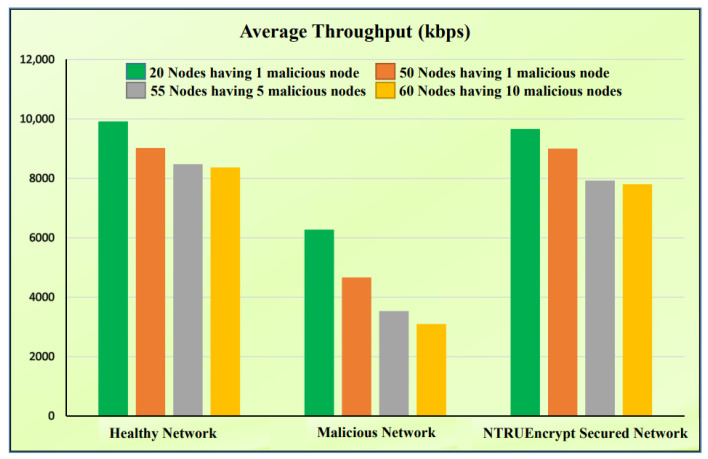
Average throughput for different node numbers.

Figure 18 shows the average throughput for varying numbers of normal and malicious nodes, as outlined below.

1. A total of 50 normal nodes and 1 malicious node.
  - Healthy network: It can be observed from Figure 18 that, in the 50-node case, the throughput decreases slightly compared to the 20-node case. This is because of the increased overhead of route discoveries. Moreover, a higher number of nodes can produce more traffic and cause channel contention, leading to collisions and retransmissions.
  - Malicious network: The figure shows that throughput decreases because the black hole-attack node still adversely affects the network. The malicious node will drop packets, but its impact might be slightly diluted in a larger network.
  - The NTRUEncrypt-secured network: The throughput decreases due to cryptographic overhead. However, this value is not far away from the healthy network. Cryptographic processing reduces the throughput, even though NTRUEncrypt cryptography ensures fewer packets are lost to the black hole-attack node compared to the malicious network.
2. A total of 50 normal nodes and 5 malicious nodes.
  - Healthy network: Figure 18 shows that throughput decreases in the 50-node scenario compared to the 20-node scenario. This is due to the increased overhead of route discoveries; the increasing node numbers cause high communication congestion and collisions.
  - Malicious network: The throughput decreases drastically compared to the 20-node malicious network case because the five black hole-attacking nodes will drop more packets, reducing the throughput.
  - The NTRUEncrypt-secured network: The throughput decreases due to the processing overhead of the NTRUEncrypt cryptography.
3. A total of 50 normal nodes and 10 malicious nodes.
  - Healthy network: Figure 18 shows that throughput decreases with the increasing number of normal and malicious nodes. This is because more nodes generate more traffic, leading to channel contention, packet collision, and retransmission.
  - Malicious network: The throughput decreases severely because the increase in the number of black hole-attack nodes causes more packet drops, degrading the throughput.
  - The NTRUEncrypt-secured network: The throughput decreases due to the computational overhead and complexities in the dense network.

#### 5.6.3. Average PDR

**Figure 19:**
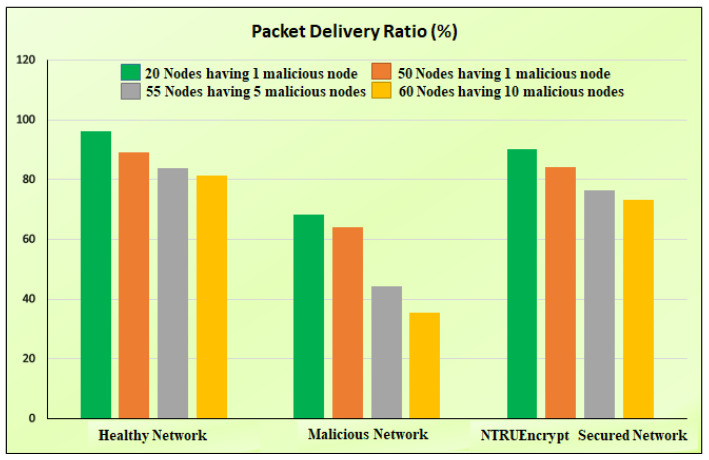
Average PDR for different node numbers.

Figure 19 shows the PDRs of the considered networks under different normal and malicious node numbers, as given below.

1. A total of 50 normal nodes and 1 malicious node.
  - Healthy network: Figure 19 shows that PDR decreases with the increase in nodes due to potential congestion and more collision chances. As the network grows denser, the potential for packet drops due to congestion increases.
  - Malicious network: The PDR decreases because the black hole node compromises more routes and drops many packets. However, its relative impact might be lessened in a more extensive network.
  - The NTRUEncrypt-secured network: The overhead from cryptography processing reduces the PDR compared to the 20-node secured network.
2. 50 normal nodes and 5 malicious nodes
  - Healthy network: Figure 19 shows that the PDR decreases with the rise in node numbers, resulting in increased communication congestion and collisions. This congestion leads to channel contention, causing more packets to be dropped.
  - Malicious network: The PDR in the 50-node scenario decreased compared to the 20-node malicious network case due to the high number of black hole-attacking nodes flooding the network and dropping more packets.
  - The NTRUEncrypt-secured network: The PDR decreases compared to the healthy network because even if the protection mechanism efficiently prevents black hole attacks, cryptographic processing and any potential inefficiencies in the mechanism lessen the PDR.
3. The 50 normal nodes and 10 malicious nodes.
  - Healthy network: Figure 19 shows that as node numbers increase, the PDR decreases. In areas of high density, the competition for medium access surges, causing packet collisions. Consequently, the PDR reduces.
  - Malicious network: Adding black hole-attack nodes intensifies the PDR reduction in the 50-node scenario compared to the 20-node scenario.
  - The NTRUEncrypt-secured network: The PDR is reduced compared to a healthy network due to cryptographic overhead and complexities in the proposed algorithm.

### 5.7. Critical Discussion

Simulation results presented in the previous subsections show that the use of the secured NTRUEncrypt routing algorithm reasonably improves network performance. Simulations have been performed by evaluating different performance metrics, such as throughput, goodput, end-to-end delay, and packet delivery ratio. Figure 15 presents the goodput of all the considered networks. It shows the goodput of a healthy network where no malicious node exists, while it also shows the goodput in the presence of a malicious node and NTRUEncrypt cryptography. It can be observed from Figure 15 that NTRUEncrypt almost achieves the same trend as depicted in the healthy case, even under a malicious node. Similarly, it can be observed from Figure 16a that NTRUEncrypt decreases the delay by 69% compared to the malicious network. However, due to cryptography and processing overhead, NTRUEncrypt increases the delay by 13% compared to a healthy network. As shown in Figure 16b, NTRUEncrypt achieves about 54% higher throughput compared to the malicious network and decreases throughput by 2.5% compared to the healthy network. Similarly, Figure 16c shows that NTRUEncrypt achieves 32% higher PDR when compared to the malicious network. This shows that the proposed system operates safely under black hole attacks, and the cryptography technique has no severe effects on system performance. Moreover, this study helps implement secure smart manufacturing for global and local industries, as shown in Table A1 and Table A2 in Appendix A, respectively.

### 5.8. Significance of the Proposed Framework and Security Methods

This subsection presents the significance of the proposed framework and security methods, as detailed in the following subsections.

#### 5.8.1. Relationships between the Proposed Framework and Security Methods

The proposed framework holistically integrates the security methods for network-based manufacturing systems. To clarify the relationships:

- Physical security: Pertains directly to the CNC machines and mobile industrial robots, ensuring that they are free from physical tampering or unauthorized access.
- Application security: It focuses on the software running on all the devices of NBMS to ensure that the applications governing their operations are free from threats.
- Network security: It focuses on secure data transmission within the NBMS while communicating remotely. Device security: It ensures that the communications of all devices of NBMS are authenticated and that data integrity is maintained.

#### 5.8.2. Enhancement or Simplification of the Achievement of Security Requirements

The proposed framework offers an approach to address the security challenges in the manufacturing industries while performing operations remotely. Integrating and collaborating mobile industrial robots with industrial machines lessen the labor force and enhance productivity. It is mandatory to tackle security concerns. The proposed framework models a real-world manufacturing environment to provide a clear visualization of how these security measures operate in tandem, allowing manufacturers to pinpoint and rectify security attacks, thus reducing complexity and simplifying the achievement of security requirements. The proposed framework also helps those industries prioritize the implementation of smart manufacturing.

#### 5.8.3. Guaranteeing Security under Personalized Customization

To guarantee the security concerns under personalized customization, the proposed framework has an inherent capacity for adaption and scalability. Whether the manufacturing industry wishes to scale up, add more devices, or adjust the roles of existing ones, the proposed framework’s modular nature allows for such adaptability. However, the underlying principles remain consistent, especially the employment of the AODV routing protocol and NTRUEncrypt cryptography. This ensures that even under various customization, the security of the system under consideration remains robust.

## 6. Conclusions

In the realm of Industry 4.0, smart manufacturing is garnering increased attention as a means to develop existing industries. Smart manufacturing systems emphasize personalized customization and stringent security measures. This study proposes a framework for a newly personalized customization factory, detailing the advanced technologies and tools used within the smart manufacturing ecosystem. To address the security measures, an IoT-enabled network-based manufacturing system with mobile industrial robots is selected as a system model to tackle the black hole attack. The NTRUEncrypt cryptography and AODV routing protocol are used to secure the system. Simulation results are presented for healthy, malicious, and secured networks. It has been found that the proposed scheme performs reasonably well for all considered performance parameters, including throughput, goodput, end-to-end delay, and packet delivery ratio, even in the presence of malicious nodes. Thus, this study serves as a foundational reference for future work, as Industry 4.0 relies heavily on personalized, secure, and efficient smart manufacturing systems. Future work will expand the framework to diverse industrial settings. Moreover, future work will seek the integration of advanced cryptographic algorithms and protocols to address emerging IoT threats. Additionally, integrating other advanced technologies, such as artificial intelligence and machine learning, could optimize the security and efficiency of smart manufacturing systems.

## Figures and Tables

**Table 1:** Comparison of different eminent-existing related works.

Authors	Technique	Attack Type	Key Findings
Jinhui et al. [31]	Intrusion detection with appropriate algorithm	Hybrid DoS attacks	Enhanced detection rate of attacking nodes, increased network lifetime, reduced attack’s impact on network traffic.
Kalkha et al. [32]	Hidden Markov Model (HMM) algorithm	Black hole attack	Showed efficient results for a secure network in terms of packet delivery ratio, end-to-end delay, and packet drop ratio.
Babaeer et al. [33]	Lightweight and secure technique	Sinkhole attack	Improved performance in packet delivery ratio, delay, energy consumption, and throughput.
Bhosale et al. [34]	Intrusion detection system (IDS)	Wormhole attack	Identified the attacker node and attack.
Ding et al. [35]	Algorithm for selective forwarding attacks	Selective forwarding attack	Reduced useless steps in density peak clustering, improved detection accuracy, false detection rate < 1%.
Hashemi et al. [36]	Backdoor technique	Penetration attack	Helped locate IoT backdoors, detecting hacker activity; prevented unauthorized access.
Ezhilarasi et al. [37]	Fuzzy- and feed-forward neural networks	Multiple network layer attacks	Average detection rate of 97.8%; high computation time.
Li et al. [38]	Privacy-preserving technique based on homomorphic encryption	Privacy intrusion	Successful prevention of privacy intrusions, computationally efficient method.
Mohsin et al. [39]	Data-driven framework	IoT threat assessment	Clear demarcation of IoT configurations and assessment of threats. Important for security concerns.
Hussein et al. [40]	Secure key management and distribution technique	Network attacks	Improved security using the elliptic curve cryptography technique and the enhanced LEACH protocol.
Goel et al. [41]	Lightweight encryption and OBT-based authentication technique (LEOBAT)	Multiple network attacks	Provided efficient and fast authentication compared to DES and blowfish.
Bilal et al. [42]	Intrusion detection model	Sinkhole attack	Exceptional results with a detection rate of 95%.
Li et al. [43]	Cluster-based algorithm	Wireless mesh	Reduced end-to-end delay and enhanced internet quality
Hammad et al. [44]	Mutual authentication and key agreement scheme	Multiple attacks	Superior performance, secure communication, resistance against attacks. Outperformed existing schemes.
An et al. [45]	Two opacity-enhancing distributed algorithms	Confidentiality in CPSs	Established necessary and sufficient conditions to ensure that a secret state was opaque and estimated accuracy maintained.
Lu et al. [46]	Privacy-preserving distributed gradient-based algorithm	Privacy preservation	Homomorphic encryption for secure multiparty computation, identification of secure computable functions based on a control-aware definition, verified correctness and computational efficiency in power system case studies

**Table 2:** Computational complexity of the AODV routing protocol.

Protocol	Phase	Step	Complexity
		Initialize the route discovery	O(n)
		Propagate the route request	O(n)
		Process the route request	O(1)
	Route Discovery	Construct the route reply	O(n)
AODV		Propagate the route reply	O(n)
		Process the route reply	O(1)
	Route maintenance	Propagate route error message	O(n)
		Process the route error message	O(1)

**Table 3:** Computational complexity of NTRUEncrypt cryptography.

Protocol	Step	Complexity
	Key generation	O(ηlogη)
NTRUEncrypt cryptography	Encryption	O(ηlogη)
	Decryption	O(ηlogη)

**Table 4:** Simulation parameters.

Parameters	Description
Platform	Ubuntu
Simulator	NS-2.35
Routing Protocol	AODV
Channel	Wireless Channel
Wireless Propagation Model	Two-Ray Ground
Area	1100 × 1100 m^2^
Simulation Time	200 s
MAC Type	Mac/802_11
Number of Mobile Nodes	20
Node speed	10 m/s
Bandwidth	3Mbps
Packet size	512 bytes
Number of Malicious Nodes	1

## Data Availability

Proposed data set is private data and will be available on request for research purposes.

## Appendix A

**Table A1:** Global Industries incorporating smart manufacturing.

Global		
**Industry**	**Company**	**Country**
	BMW	Germany
Automotive	Ford Motor	United States
	Toyota Motor Corporation	Japan
	Siemens	Germany
Electronic	Apple Inc.	United States
	Samsung Electronics Co. Ltd	South Korea
	PepsiCo	United States
Food and Beverage	Nestle S.A	Switzerland
	The Coca-Cola	United States
	Airbus	France
Aerospace	Boeing	United States
	Northrop Grumman Corporation	United States
	Pfizer Inc.	United States
Healthcare	Johnson & Johnson	United States
	Novartis International AG	Switzerland

**Table A2:** Local industries incorporating smart manufacturing.

Local (Pakistan)		
**Industry**	**Company**	**City**
	Honda Atlas Cars (Pakistan) Limited	Kasur
Automotive	Pak Suzuki Motor Company Limited	Rawalpindi
	Indus Motor Company Limited (Toyota)	Karachi
	Nishat Mills Limited	Lahore
Textile	Gul Ahmed Textile Mills Limited	Karachi
	Faisal Spinning Mills Limited	Karachi
	Nestle Pakistan Limited	Lahore
Food and Beverage	Engro Foods Limited	Karachi
	Mitchell’s Fruit Farms Limited	Lahore
	Packages Limited	Islamabad
Plastic	Engro Polymer & Chemicals Limited	Karachi
	Master Group of Industries	Lahore
	GlaxoSmithKline Pakistan Limited	Karachi
Healthcare	Searle Pakistan Limited	Karachi
	LCI Pakistan Limited	Karachi
